# Cinobufagin exerts an antitumor effect in non-small-cell lung cancer by blocking STAT3 signaling

**DOI:** 10.7150/jca.86544

**Published:** 2023-10-02

**Authors:** Sunshun Yan, Chunbo Ma, Feng Zhou, Hailun Zheng, Lehe Yang, Zhongxiang Xiao, Jiandong Zhu, Haiyang Zhao, Chengguang Zhao, Xiaoling Xu

**Affiliations:** 1 Cheeloo College of Medicine, Shandong University, 44 Cultural West Road, Shandong Province 250012, China.; 2 The First Affiliated Hospital of USTC, Division of Life Sciences and Medicine, University of Science and Technology of China, Hefei, Anhui 230001, China.; 3 The Second Affiliated Hospital and Yuying Children's Hospital of Wenzhou Medical University, Wenzhou, 325027, China.; 4 The Institute of Life Sciences, Wenzhou University, Wenzhou, Zhejiang 325035, China.; 5 Affiliated Yueqing Hospital, Wenzhou Medical University, Wenzhou, Zhejiang 325600, China.

**Keywords:** Non-small-cell lung cancer, Cinobufagin, STAT3

## Abstract

**Background:** Non-small-cell lung cancer (NSCLC) is the most common histological subtype of lung cancer with significant morbidity and mortality rates worldwide. Cinobufagin, the primary component of Chansu and the major active ingredient of cinobufacini, has attracted widespread attention for its excellent anticancer effects, but its activity remains poorly characterized in NSCLC.

**Methods:** The functions of cinobufagin treatment in anti-tumor was evaluated using various in vitro and in vivo assays. The change of STAT3 signaling by cinobufagin was analyzed using molecular docking, immunofluorescence technic and western blotting.

**Results:** In vitro, we confirmed the inhibitory effect of cinobufagin on cell viability, proliferation, migration, epithelial-mesenchymal transition (EMT), as well as an apoptosis-inducing effect. The antitumor effects of cinobufagin were confirmed in vivo by measuring tumor growth in a mouse xenograft model. Cinobufagin was found to significantly inhibit the phosphorylation of signal transducer and activator of transcription 3 (STAT3) at tyrosine 705 (Y705) in a time- and concentration-dependent manner. Moreover, cinobufagin reversed IL-6-induced nuclear translocation of STAT3.

**Conclusions:** Our study has demonstrated that cinobufagin exerts an antitumor effect in non-small-cell lung cancer by blocking STAT3 signaling, and cinobufagin is a promising candidate agent for NSCLC therapy.

## Introduction

Non-small-cell lung cancer (NSCLC) is the dominant histological subtype of lung cancer and one of the most common types of cancer globally 1, 2. Clinically, surgical resection remains the preferred treatment for early-stage NSCLC. However, most patients are diagnosed at an inoperable stage due to insidious symptoms and late diagnosis 3. Recent advances in immunotherapy and targeted therapy have led to improved prognosis in some patients with NSCLC; however, treatment resistance and subsequent relapse remain a major concern 4. The five-year survival rate of NSCLC ranges from 10% to 20% in most countries 1, 5. Thus, more effective therapeutic strategies are urgently required to improve the clinical outcomes of NSCLC.

Signal transducer and activator of transcription 3 (STAT3)-a core regulator of the STAT family that is overexpressed in various cancers including NSCLC-has become an attractive molecular target for cancer therapy 6. STAT3 phosphorylation at the tyrosine 705 (Y705) residue and nuclear accumulation of P-STAT3 are two dominant indicators of STAT3 activation. Activation of the STAT3 signaling pathway is associated with cancer cell survival, proliferation, immunosuppression, angiogenesis, invasion, and metastases, among other tumorigenic processes 7-10. In NSCLC, poor prognosis and chemotherapy resistance were found to be related to the persistent activation of STAT3, and the inhibition of STAT3 activity can effectively inhibit tumor progress 11-13. Because of this association, STAT3 is considered a promising candidate target for cancer treatment. However, no STAT3-specific inhibitor has been approved for clinical use 14.

Chansu, known as Venenum Bufonis, is a traditional Chinese medicine extracted from the defensive secretions of *Bufo gargarizans* Cantor and *Duttaphrynus melanostictus* 15. Cinobufacini (Huachansu), an intravenous preparation originating from toad skin, has been widely used in cancer therapy and hepatitis B virus treatment 16, 17. As the primary component of Chansu and the major active ingredient of cinobufacini, cinobufagin exhibits potent antitumor effects in various cancers 18-25. In our earlier study, we confirmed that cinobufagin can inhibit epithelial-mesenchymal transition (EMT) via STAT3 signaling in colorectal cancer 23. Recently, Zhang et al. 26 reported that cinobufagin induced FOXO1-regulated apoptosis, proliferation, migration, and invasion in NSCLC. However, the antitumor effects and precise cellular mechanisms of cinobufagin in NSCLC remain poorly characterized.

In this study, we investigated the antitumor effects and cellular mechanisms of cinobufagin in NSCLC. Our results suggest that cinobufagin can inhibit the STAT3 signaling pathway in NSCLC, thereby inducing cell apoptosis and suppressing cell proliferation, migration, and tumor growth (in an NSCLC xenograft model). Thus, cinobufagin is a promising candidate for NSCLC therapy.

## Materials and methods

### Antibodies and reagents

Cinobufagin (HPLC ≥98%) was purchased from Baoji Herbest Bio-Tech Co., Ltd. (Baoji, Shaanxi,China). Napabucasin was purchased from MedChem Express LLC (Monmouth Junction, NJ, USA).Both cinobufagin and napabucasin were dissolved in dimethyl sulfoxide (DMSO) at a concentration of 20 mM, aliquoted, and stored at -80 °C for subsequent use. DMSO and methylthiazolyldiphenyltetrazolium bromide (MTT) were provided by Sigma-Aldrich Co. (St. Louis, MO, USA). The antibodies against STAT3 (phospho Y705), BCL2, BAX, and LMNB1, the horseradish peroxidase (HRP)-conjugated goat anti-rabbit IgG and goat anti-rabbit IgG H&L (Alexa Fluor® 555), were purchased from Abcam (Cambridge, UK). The antibodies against STAT3, Vimentin, MMP-2, Ki67, and GAPDH and the HRP-conjugated horse anti-mouse IgG were supplied by Cell Signaling Technology Inc. (Danvers, MA, USA). Total Protein Extraction Kit (AR0103, AR0101) was supplied by Boster Biological Technology (Wuhan, China). The Bradford protein-assay kit used in this study was obtained from BioRad Laboratories Inc. (Hercules, CA, USA). The fluorescein isothiocyanate (FITC) Annexin V Apoptosis Detection Kit I (Pharmingen (556547)) was purchased from BD Pharmingen (Franklin Lakes, NJ, USA). The EdU incorporation assay kit was obtained from RiboBio Co., Ltd. (Guangzhou, China). Fetal bovine serum (FBS), phosphate-buffered saline (PBS), 1% penicillin/streptomycin, and all the culture media were purchased from Thermo Fisher Scientific Inc. (Waltham, MA, USA).

### Cell lines and culture

Human NSCLC cell lines PC-9 and H460, purchased from the Shanghai Institute of Biosciences and Cell Resources Center (Chinese Academy of Sciences, Shanghai, China), were cultured in RPMI-1640 or DMEM media (Thermo Fisher Scientific) with 10% FBS (Thermo Fisher Scientific) and 1% penicillin-streptomycin (Solarbio). Both cells were maintained in a humidified cell incubator at 37 °C with 5% CO_2_.

### MTT cytotoxicity assay

Human NSCLC cells (H460 and PC9, 4×10^3^ cells/well) in their logarithmic growth phase were seeded in each well of a 96-well plate and incubated with fresh medium containing different concentrations of cinobufagin at 37°C under 5% CO_2_ for 48h. Six multiple holes are established for each concentration. Subsequently, MTT (25 μL/well) was added and incubated for 4 h at 37 °C. The MTT solution was then discarded, and 150 μL DMSO was added into each well to dissolve formazan crystals. After full shaking, the optical density of each well at 490nm (OD 490nm) was measured and used to calculate the IC50 value via GraphPad Prism (version 9.0).

### Clonal assay

Suspensions of single NSCLC cells were prepared and seeded in 6-well plates with a density of 500 cells/well. The cells were treated with the specified concentration of cinobufagin for 24 h and cultured at 37°C under 95% CO2 for 7 d. The single-cell clone images were captured after crystal violet staining and PBS washing.

### EdU cell proliferation detection

Cells were seeded in 6-well plates at a density of 2×10^5^ cells/well and were treated with cinobufagin of designed concentrations (0.1, 0.2, and 0.5 μM) for a specified time. Then, Edu was added at a final concentration of 10 μM and incubated for 2h before detection. Subsequent click reaction and Hoechst 33342 staining were conducted according to the instructions of the EdU Cell Proliferation Kit with Alexa Fluor 555(BeyoClick™). The number of EdU-labelling cells was determined by fluorescence microscopy and counted with Image J (version 1.48). The positive cells' rate was calculated by GraphPad Prism (version 9.0).

### Immunofluorescence staining (IF) Microscopy

For the Immunofluorescence staining, cells inoculated in fluorescence cuvettes were fixed with 4% paraformaldehyde, permeabilized using 0.5% Triton X-100 (in PBS) and washed 3 times with PBS. Cell slides were blocked with 1% bovine serum albumin (BSA) in PBS at 37°C for 30 min followed by primary antibodies (Ki67, P-STAT3, Vimentin) incubation at 4°C overnight. After being washed with PBS, Cell slides were then incubated with relative secondary antibodies (Alexa Fluor goat anti-mouse IgG antibody or Alexa Fluor 488-labeled anti-rabbit IgG antibody) at room temperature for 60 min, followed by 15 min 4',6-diamidino-2-phenylindole (DAPI) stain. Anti-fluorescence quenching sealing tablets were used to prevent fluorescence quenching until they were captured by confocal microscopy.

### Immunohistochemical analysis (IHC)

The tumor tissues from NSCLC xenograft model were fixed at room temperature in 4 % para formaldehyde and embedded in paraffin. Paraffin-embedded tissue with a thickness of 5μm were prepared, deparaffinized, subjected to antigen retrieval with citric acid at 98 ℃ for 5 min, and incubated with primary antibodies (Ki67 and Cleaved caspase3) at 4℃ overnight. The signaling was marked by incubating with the corresponding biotinylated secondary antibody for 60min, then staining with VULCAN FAST RED CHROMOGEN kit2 for 15min at room temperature. Thereafter, the expression of the target proteins was visualized using diaminobenzidine and counterstained with hematoxylin. The scoring criteria were described as previously. The staining results were assessed based on the percentage of positive cells and the staining intensity. The proportions of positive cells were assigned scores of 0 (0-5 %), 1(5-25 %), 2 (25-50 %), 3 (50-75 %) or 4 (75-100 %). The staining intensity was assigned the scores of 0 (no staining), 1 (light yellow), 2 (yellow), or 3 (brown). The staining intensity values were multiplied by the proportion to obtain an individual score for each specimen.

### Cell apoptosis assay

NSCLC cells were seeded in 6-well plates for overnight attachment, stimulated with cinobufagin of designed concentrations, and harvested using trypsin solution without EDTA. Subsequently, Annexin V and propidium iodide incubation was launched under the guidance of the descritption of the FITC Annexin V Apoptosis Detection Kit. The sample was analyzed on the BD FACSCalibur platform (BD Biosciences, Baltimore, MD, USA).

### Molecular Docking

The protein structure of STAT3 in complex with a small molecule named SD-36 reported by Bai et al. was retrieved from the Protein Data Bank (PDB code: 6NJS) for molecular docking study. The fastDRH server 27 was used to predict the binding mode between STAT3 and cinobufagin with the AutoDock Vina docking engine^28^. The coordinates of SD-36 extracted from the crystal structure was uploaded as pocket reference. The maximum number of binding modes to generate was set to ten. Finally, the pose with the best binding score was used for the subsequent analysis.

### Western blot analysis (WB)

Total protein samples, extracted from cells under different treatments, were quantified by Coomassie Brilliant Blue staining. Configured samples were heated at 100 ◦C for 10 min, separated by SDS-PAGE gels, and transferred onto PVDF membranes. 1.5 h 5% fat-free milk incubation was conducted to block nonspecific sites, and then the membranes were washed with Tris Buffered Saline with Tween 20 (TBST). The related blots were incubated with specific primary antibodies, followed by secondary antibodies the next day. western blot images were analyzed with Image J (version 1.48).

### Wound-healing assay

Cells were seeded in 6-well plates at a density of 5×10^5^ cells per well and cultured until wells reached approximately 100% confluence. Three straight scratches were made in each well using sterilized 10 μl pipette tips. Then treat the cells with designed concentrations of cinobufagin. The scratches were photographed at 0, 24, and 48 hours.

### Invasion assay

200 µL NSCLC cell suspensions in serum-free medium were prepared and added to each transwell chamber, which was placed in the well of a 24-well plate containing serum-free medium, for overnight attachment. The serum-free medium in a 24-well plate was then replaced with medium containing 10% FBS, the cinobufagin treatment was launched at the same time. After 12-24h, the chambers were removed and stained with crystal violet. The chambers were photographed after the non-invading cells were carefully wiped off with cotton swabs.

### Cytoplasmic and Nuclear Protein Extraction

H460 cells were seeded in a 100 mm dish, allowed to attach overnight, and treated with different concentrations of the indicated drugs. Cells were stimulated with IL-6 for 30 min before being lysed. The cytoplasmic and nuclear proteins were isolated by following the nuclear and cytoplasmic extraction kit protocols. The cytoplasmic and nuclear protein levels were determined by Western blot analysis.

### In Vivo Tumor Xenograft Mouse Model

The female nude mice (BALB/c nude, 4-5-week-old) used in this study were obtained and housed by the Laboratory Animal Center of Wenzhou Medical University under sterile specific pathogen-free conditions. To build a subcutaneous NSCLC xenograft mouse model, H460 cells were collected and resuspended in a 1:1 solution of PBS and Matrigel matrix, at a concentration of 5 × 10^6^/mL. Cell suspensions were subcutaneously injected in the right flank of mice, and after 7 days the mice were randomly allocated into four groups: vehicle group (n = 7), cinobufagin 1 mg/kg (n = 7), cinobufagin 2 mg/kg (n = 7), and napabucasin (Napa) 10 mg/kg (n = 7). Drugs were administered intraperitoneally every 2 days. We used a sliding caliper to measure tumor size every 2 days and calculated the tumor volume using the following formula: volume = 0.5 × length × width. On day 14, the nude mice were sacrificed, and the heart, liver, kidneys, and lungs were collected and fixed in 4% paraformaldehyde for hematoxylin and eosin (H&E) staining. The tumors were dissected, photographed, weighed, and preserved at -80 °C. All animal experiments were conducted under the guidance of the institutional ethics and safety guidelines (Institutional Animal Welfare and Ethics Committee, Wenzhou Medical University, Wenzhou, Zhejiang, China).

## Results

### Cinobufagin negatively affects the viability and proliferation of NSCLC cells

**Figure 1:**
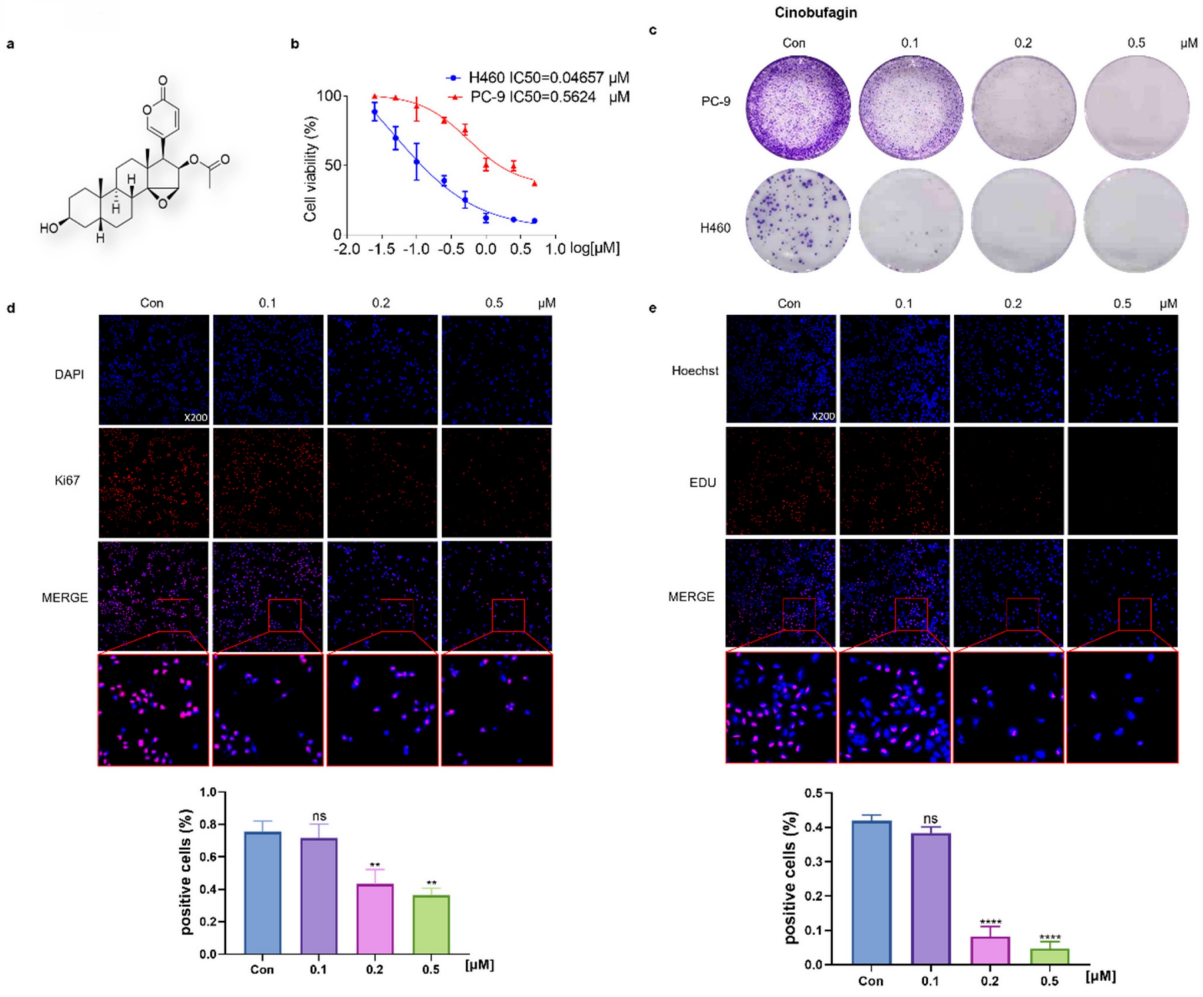
Cinobufagin inhibits the proliferation of NSCLC cells. (a) Chemical structure of cinobufagin. (b) Cells treated with the drug at different concentrations for 48 h; formation of blue formazan crystals (solubilized with dimethylsulfoxide) by cells incubated with the MTT solution; absorbance values at OD490 nm. (c) NSCLC cells at three concentrations (0.1, 0.2, and 0.5 µM) incubated using cinobufagin for 12-20 h until the appearance of colonies; cells stained with crystal violet. (d) Changes in the signals received by proteins (Ki67) in PC-9 were verified by immunofluorescence staining; and the satatistical analysis of the imunofluorescence staining data. (e) Proliferation of PC-9 were detected using BeyoClick™ EdU-555 (red) immunofluorescence staining kit; and the satatistical analysis of the imunofluorescence staining data. Capacity of cinobufagin to suppress NSCLC cells, as determined from the four aforementioned experiments. (**P < 0.01, ****P < 0.0001).

To evaluate the effectiveness of cinobufagin, a bioactive component originating from Bufo bufo gargarizans (Figure 1a), the MTT assay was launched first. The results showed that half-maxima concentration values (IC50) of NSCLC cells (PC-9 and H460) were 0.5624 μM and 0.04657 μM (Figure 1b), which confirms its extraordinary inhibitory effect on NSCLC cell viability. Based on the IC50, a concentration gradient (0.1, 0.2, and 0.5 μM) was designed for the following experiments. Then, the clonal assay exhibited a concentration-dependent inhibitory effect on NSCLC cells' colony formation capacity (Figure 1c). Overall, these results suggest that cinobufagin effectively reduced the viability of the NSCLC cells. To further explore the effects of cinobufagin on NSCLC cell proliferation, Ki67 immunofluorescence and EdU cell proliferation assay were applied. The results show cell proliferation marker (Ki67) staining and the EdU fluorescent label were significantly decreased (Figure 1d-e) with increased drug concentration. Overall, we confirmed that cinobufagin restrains the viability and proliferation of NSCLC cells.

### Cinobufagin induces apoptosis in human NSCLC cells

**Figure 2:**
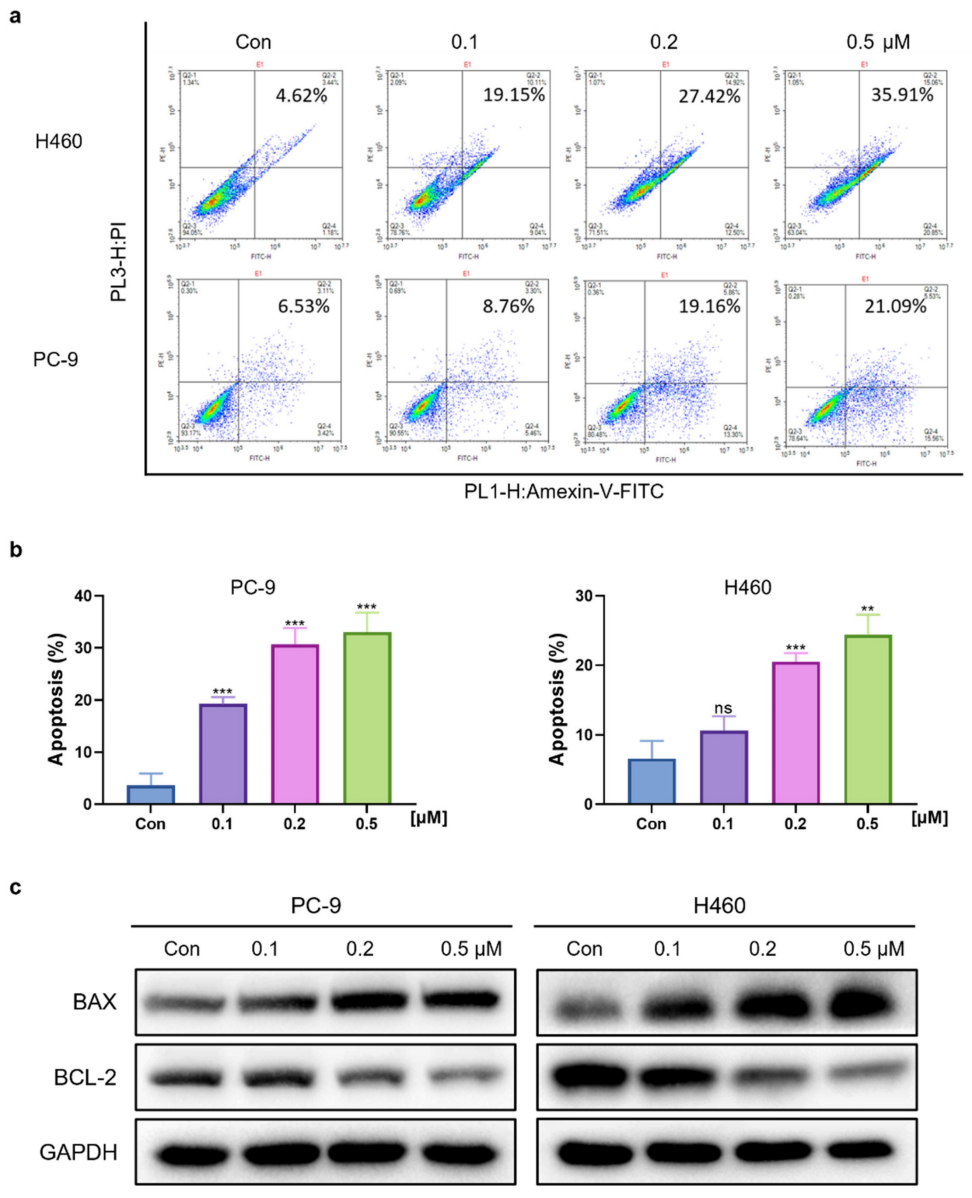
Cinobufagin promotes the apoptosis of human NSCLC cells. (a) NSCLC cells treated with cinobufagin for 20-24 h; apoptotic cells were treated using the Annexin V-FITC apoptosis kit, and the percentage of cell apoptosis as analyzed by flow cytometry. (b) Statistical analysis of the flow cytometry analysis data. (c) BCL-2 and BAX proteins extracted from cells lysed with protease inhibitors after treatment for 20-24 h, using GAPDH as the internal reference. Representative images shown were chosen from three independent experiments. **P < 0.01, ***P < 0.001.

To determine whether the cinobufagin treatment can induce cell apoptosis in NSCLC, cell apoptosis assay was launched via flow cytometry and the FITC Annexin V Apoptosis Detection Kit. The results showed that the proportion of apoptotic cells was significantly increased along with the concentration of cinobufagin, which revealed the proapoptotic effect of cinobufagin in NSCLC cells (Figure 2a-b). Furthermore, the expression of apoptosis-related proteins, Bcl-2 and Bax were detected using western blot analysis. As the dose of cinobufagin increased, the expression of Bax was increased while Bcl-2 was decreased (Figure 2c). Overall, we proved that cinobufagin can induce apoptosis in human NSCLC cells.

### Cinobufagin inhibits NSCLC cells migration and EMT

**Figure 3:**
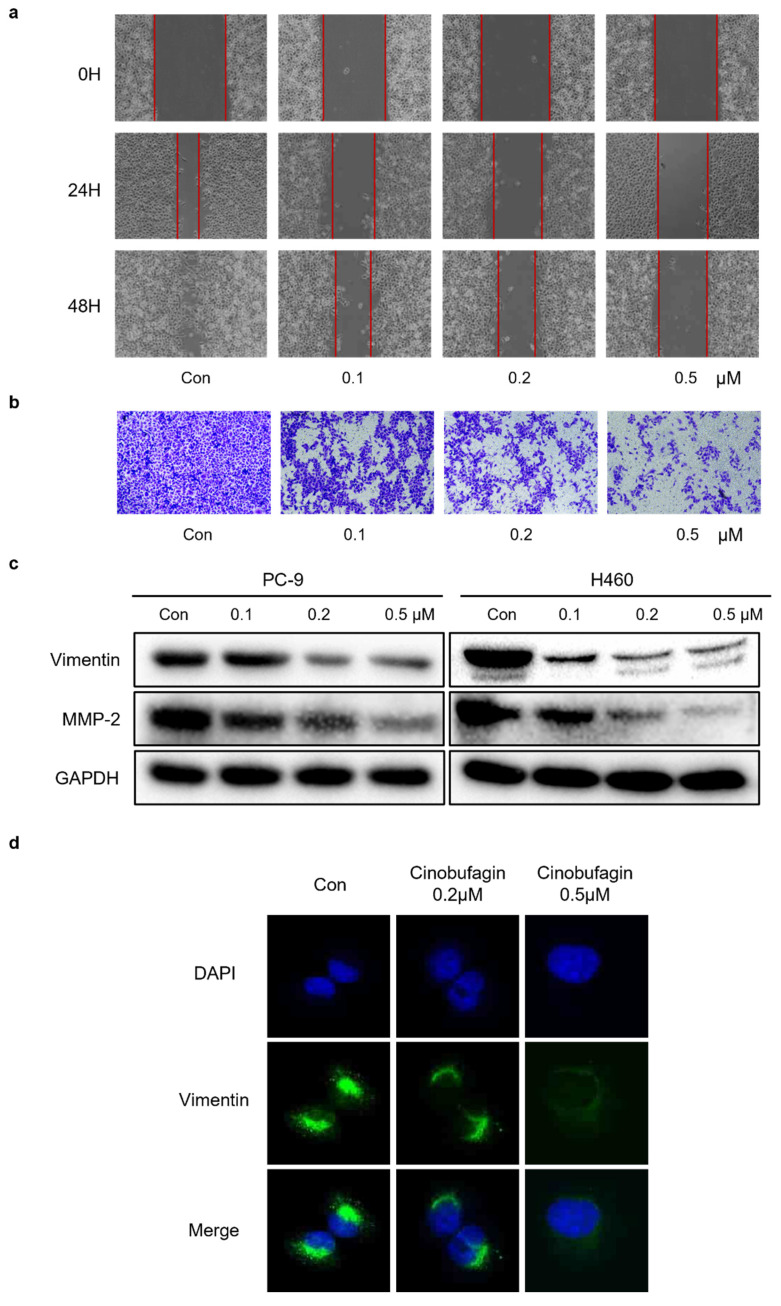
Cinobufagin inhibits the invasion and metastasis of NSCLC cells. (a) Wound healing assay of PC-9 cell. (b) PC-9 incubated on the upper side of the chamber and treated with cinobufagin for 10-20 h; cells stained with crystal violet to estimate the invasion ability of NSCLC cells. (c) Expression of proteins related to EMT signaling pathways as detected by western blot analysis. (d) Changes in the signals received by proteins (Vimentin) in PC-9 cell were verified by immunofluorescence staining.

To demonstrate whether cinobufagin can affect the migration capability of NSCLC cells, wound-healing assay and transwell migration assay were applied. The results uncovered that the wound healing rate of PC-9 cells was remarkably reduced in the cinobufagin-treated groups compared to the control group (Figure 3a), while the proportion of migrating NSCLC cells was also decreased significantly in a concentration-dependent manner (Figure 3b). To sum up, cinobufagin has a considerable inhibitory effect on cell migration abilities. The activation of epithelial to mesenchymal transition (EMT) plays a pivotal role during the tumor progression, which results in tumor metastasis and drug resistance 29-31. We assessed the effect of cinobufagin on the EMT of NSCLC by analyzing the expression of EMT-associated proteins via western blot analysis and immunofluorescence staining. We found the increasing concentration of cinobufagin treatment could decrease Vimentin and MMP-2 expression (Figure 3c), while the immunofluorescence staining also indicated decreased fluorescence of vimentin compared to the control group (Figure 3d). Therefore, we conclude that cinobufagin not only inhibited NSCLC cell migration, but also inhibited the EMT progress.

### Cinobufagin exerts antitumor effect via blocking STAT3 signaling

**Figure 4:**
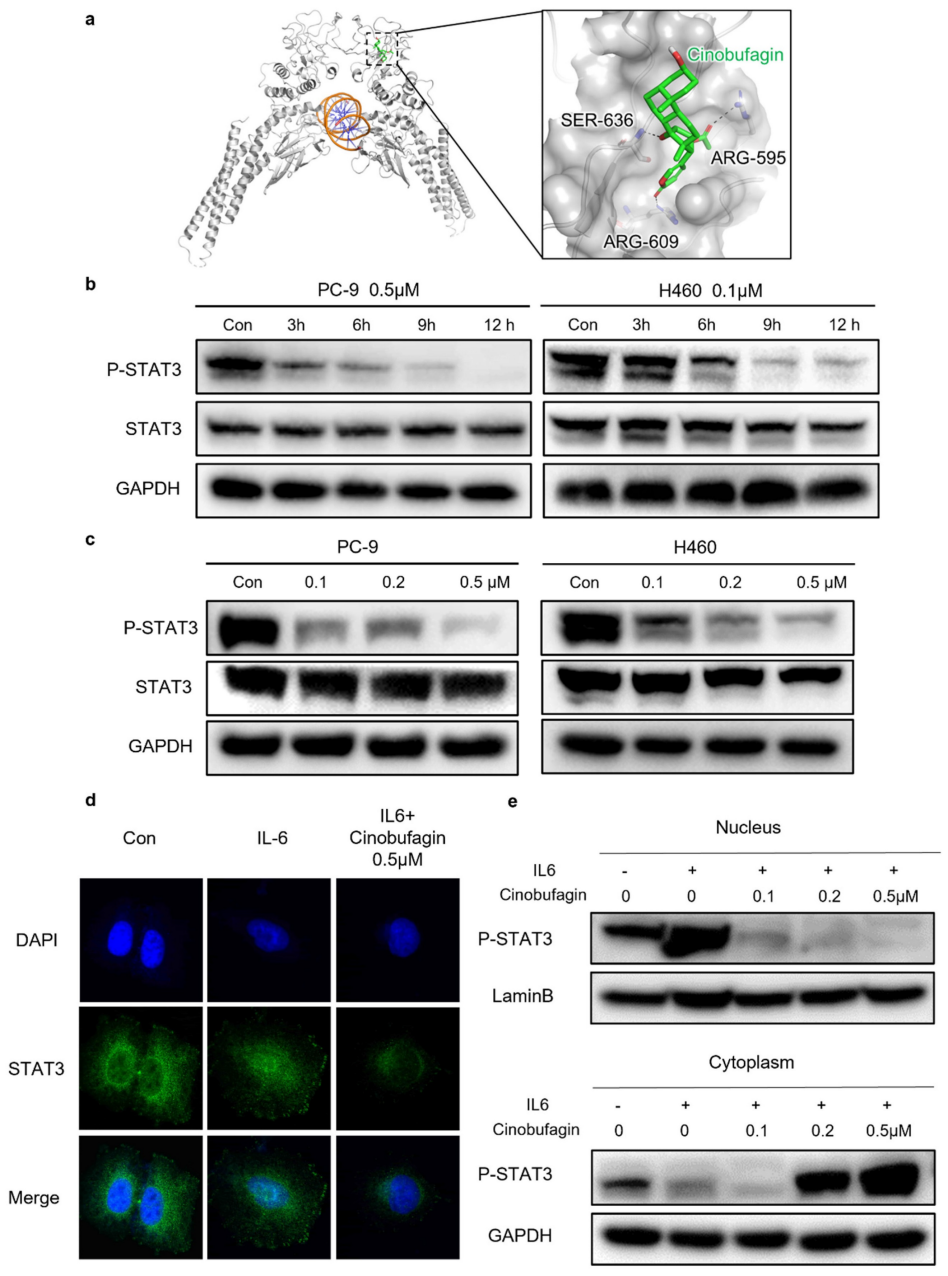
Cinobufagin inhibits STAT3 signaling. (a) Predicted binding mode between STAT3 and cinobufagin. Hydrogen bond is colored red. (b-c) Expression variation of proteins related to STAT3 signaling pathways in response to cinobufagin treatment with different concentration-time as detected by Western blot analysis. (d) Cellular localization of proteins as visualized by immunofluorescence staining in PC-9; analysis of changes in the intracellular location of STAT3. (e) Nuclear and cytosolic proteins extracted from PC-9 after treatment; distribution of STAT3 in cells as determined by western blot analysis.

Even though there have been several reports on the anti-tumor effect of cinobufagin achieved by several signaling pathways, the precise mechanism of cinobufagin in NSCLC has not yet been elucidated thoroughly. As shown in Figure 4a, we investigated interactions between the SH-2 domain of STAT3 and cinobufagin by molecular docking analysis. The docking results revealed that the cinobufagin occupied three solvent-accessible sub-pockets of SH-2 domain. In addition, the cinobufagin formed three bonds with the residues of SER-636, ARG-595 and ARG-609 to stabilize the conformation (Figure 4a).

Therefore, further studies investigating the precise relationship between cinobufagin and STAT3 signaling in NSCLC were conducted through western blot analysis, which suggests cinobufagin treatment didn't suppress the expression of STAT3 itself, but the effect of cinobufagin treatment on inhibiting STAT3 phosphorylation was increasing with time and concentration (Figure 4b-c). The nuclear translocation of phosphorylated STAT3 acted as another feature of the activation of the STAT3 signal. Based on the above results, we hypothesized that cinobufagin could reverse IL-6-induced nuclear translocation of P-STAT3, the immunofluorescence staining for STAT3 phosphorylation in NSCLC cells shows that most P-STAT3 was primarily localized to the cytoplasm and then translocated to the nucleus in response to IL-6 stimulation. Then, the cinobufagin treatment reverse this process and decreased the expression of P-STAT3 as well (Figure 4d). Similar results were obtained via nuclear/cytoplasmic isolation and relative western blot analysis (Figure 4e). Overall, our above results strongly suggest that cinobufagin can block STAT3 signaling by disturbing STAT3 phosphorylation and nuclear translocation in a time and concentration-dependent manner.

### Cinobufagin inhibits tumor growth in human NSCLC xenograft model

**Figure 5:**
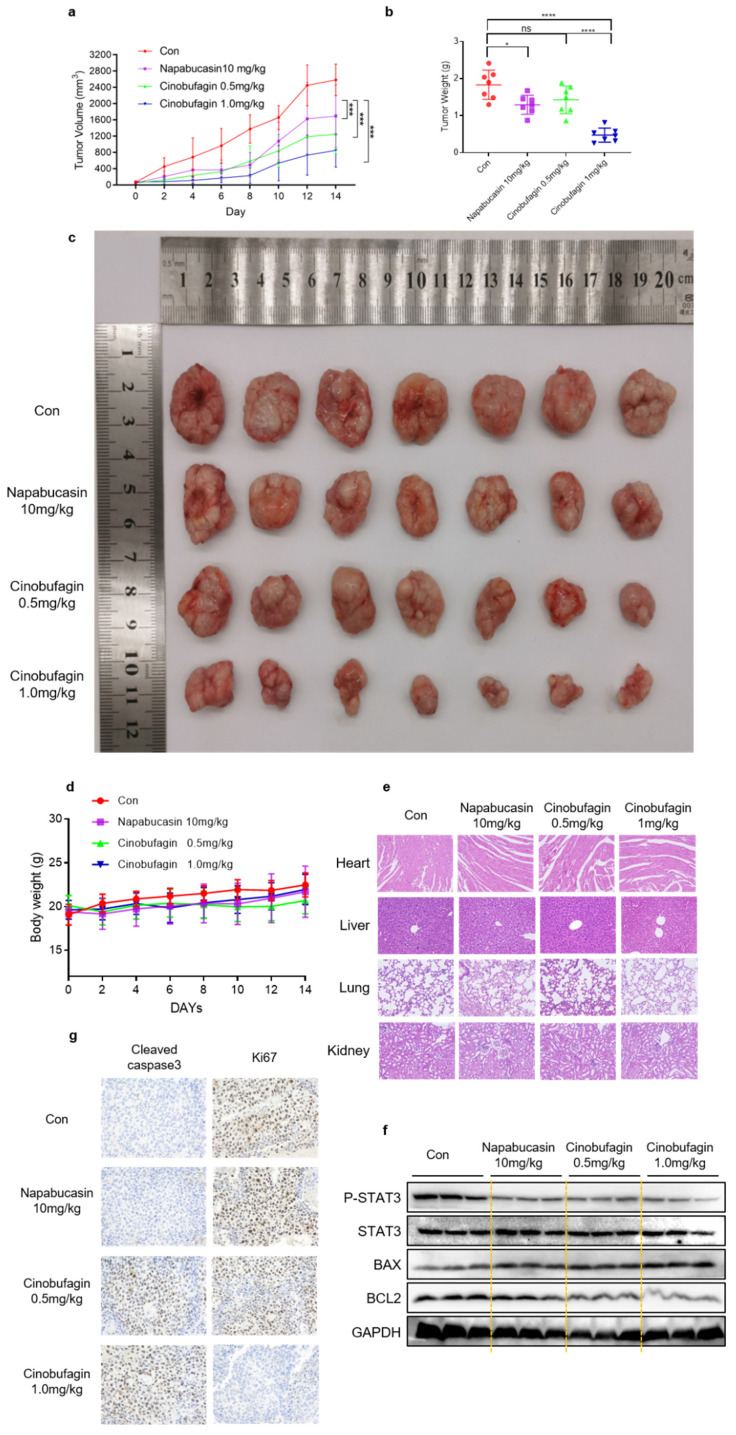
Cinobufagin inhibits the growth of NSCLC xenograft models. (a) Nude mice injected with napabucasin (10 mg/kg) or cinobufagin (0.5 or 1.0 mg/kg) via the intraperitoneal route every other day for 7 doses, and measured the tumor volumes. (b) Comparison of the tumor weights of the four groups. (c) Size of the xenograft tumor (by direct observation). (d) Weight changes in mice during 14 d. (e) Hematoxylin-eosin staining of the heart, liver, lungs, and kidneys to check for toxicity. (f) Immunohistochemical staining images of cell proliferation marker Ki-67 and Cleaved Caspase 3 in tumor tissues. (g) Homogenized fresh tumor tissues; Expression of STAT3- and apoptosis-related proteins detected by western blot analysis (*P < 0.05, ***P < 0.001, ****P < 0.0001).

Furthermore, the therapeutic efficacy of cinobufagin *in vivo* was investigated via human NSCLC tumor xenograft models established by subcutaneously injecting H460 cells into nude mice. Longitudinal tumor monitoring revealed that cinobufagin treatment inhibited tumor growth significantly compared to the vehicle group and positive control group (Figure 5a). All these resected tumor weights statistics and representative tumor graphs are presented in Figure 5b-c. Moreover, no significant body weight loss was detected in any group during the treatment (Figure 5d), while the H&E staining shows no noticeable toxicity on the major organs (heart, liver, lung, and kidneys) at this concentration (Figure 5e). All these results suggest that no obvious treatment-related toxicity was observed. To further investigate the *in vivo* anti-tumor mechanism of cinobufagin, western blot analysis of tumor tissue extracts was executed. The results uncovered that cinobufagin downregulated the levels of P-STAT3^Y705^ and BCL2, and enhance the expression of BAX, which are generally consistent with the in vitro experiments (Figure 5f). Additionally, the down-regulated Ki67 and up-regulated Cleaved caspase 3 expression in tumor tissue compared to the vehicle group and positive control group was determined by immunohistochemistry (Figure 5g). Overall, our findings indicated that cinobufagin exerts significant antitumor effects in NSCLC in vitro and in vivo via inhibiting STAT3 signaling with no obvious treatment-related toxicity, and cinobufagin is a promising candidate agent for NSCLC therapy.

## Discussion

Natural products, the main sources of traditional Chinese medicine with a long and rich history in cancer therapy, have been a unique resource for the discovery of antitumor ingredients 32. Cinobufagin, the primary component of Chansu and the major active ingredient of cinobufacini, exhibits significant antitumor effects in diverse cancer 18-25. Previous study have reported that cinobufagin induced FOXO1-regulated apoptosis, proliferation in A549 cells and mice model 26, and cinobufagin inhibited proliferation, migration and invasion of H1299 cells and Wnt/β-catenin, Src/STAT3 and FAK/Src signaling pathways were probably involved in this antitumor effect 33. These studies draw a conclusion with one NSCLC cell lines. To increase representation of the selected cell lines, we further investigated the antitumor effect of cinobufagin with two other NSCLC cell lines (PC-9 and H460). Although Zhang et al. also suggested that cinobufagin suppressed the progression of four NSCLC cell lines through AKT signaling pathway 34, they did not identify this action in vivo which were further studied in our work. In this study, we confirm the strong antitumor activity of cinobufagin in the NSCLC cell line and xenograft model, then verified the anticancer mechanism. Although the antitumor activity of cinobufagin has been widely recognized in the literature, its effect in NSCLC cell lines still remain poorly described. So, we sought to assess the effect of cinobufagin on the proliferation, apoptosis and migration, three vital physiological processes of two NSCLC cell lines. In our MTT study, the lowest IC50 reached nanomolar level. Furthermore, cinobufagin show a significant suppression of the colony formation capacity and proliferative activity of NSCLC cell in clonal assay and EdU assay. We also identified the cinobufagin treatment does downregulate the expression of Ki67, a well-recognized proliferation marker 35, in NSCLC cells via immunofluorescence staining. As for the apoptosis and migration, flow cytometry analysis of apoptosis shows increased apoptotic rate in both cell lines and further analysis of the apoptosis relevant protein Bcl-2 and Bax come up with same conclusion. Meanwhile, the transwell migration assay and wound-healing migration assay indicated a considerable inhibitory effect on cell migration abilities. Combined with characterization experiments, the decreased expression of Vimentin and MMP-2 indicated the anti-EMT effects of cinobufagin in NSCLC^36^, ^37^. Similar changes in protein expression were obtained in the tumor tissue from the xenograft model. Altogether, cinobufagin does exhibit profound anti-tumor activity in NSCLC.

Different from previous relevant studies, we reported the important role of STAT3 signaling in the antitumor activity of cinobufagin, and proposed the potential of cinobufagin to serve as a safe and effective inhibitor of STAT3 for NSCLC therapy. JAK/STAT3 pathway, mainly overactivated by cytokine, hormone, and growth factor, has become an attractive target for cancer intervention^8^, ^13^. STAT3 mediates the expression of a variety of genes in response to cell stimuli and serves a promote oncogenesis enhancing cancer cell proliferation, migration, and survival^6^. According to our previous research, aberrant STAT3 activation is involved in abnormal proliferation and resistance to apoptosis of NSCLC cells^38^. Additionally, an active IL-6R/STAT3/miR-34a loop was detected to play an important role in EMT^36^.

By inhibiting STAT3 phosphorylation and nuclear translocation, cinobufagin could transcriptionally regulate STAT3 activity, which could inhibit the occurrence and development of tumor. However, no STAT3-specific inhibitor has been approved for clinical use to date^14^. The discovery of active pharmaceutical ingredients with anti-STAT3 property from natural products has become a new direction for researchers. Our study confirms that cinobufagin could inhibit STAT3 phosphorylation in tyrosine 705, and IL6-induced nuclear translocation in vitro, which proves the inhibitory effect of cinobufagin in STAT3 activation. The protein extracted from tumor tissue shows a similar result. These novel findings may aid the proper interpretation of the molecular mechanisms of cinobufagin in anti-NSCLC treatment. Based on the research mentioned above, we will further study its pharmacokinetic and toxicological information. We will also work on its structural optimization and dosage form optimization, combine cinobufagin with nano-drug delivery system, to promote it clinical translation.

Overall, we speculate that cinobufagin is a natural active ingredient with antitumor activity, which may exert an antitumor effect in non-small-cell lung cancer by blocking STAT3 signaling. In addition, therapeutic concentrations of the cinobufagin exhibited considerable effects without damaging the organ, which suggests the potential of cinobufagin to serve as a safe and effective inhibitor of STAT3 for NSCLC therapy.

## Supplementary Material

Supplementary figures.
