# Supplementary material for: Cinobufagin exerts an antitumor effect in non-small-cell lung cancer by blocking STAT3 signaling
Source: J Cancer. 2023 Oct 2;14(17):3309–20. doi: 10.7150/jca.86544 (PMC10622998; doi:10.7150/jca.86544)
Supplement: Supplementary file 1 — Supplementary figures. [file jcav14p3309s1.pdf]

Supplementary Figure 1

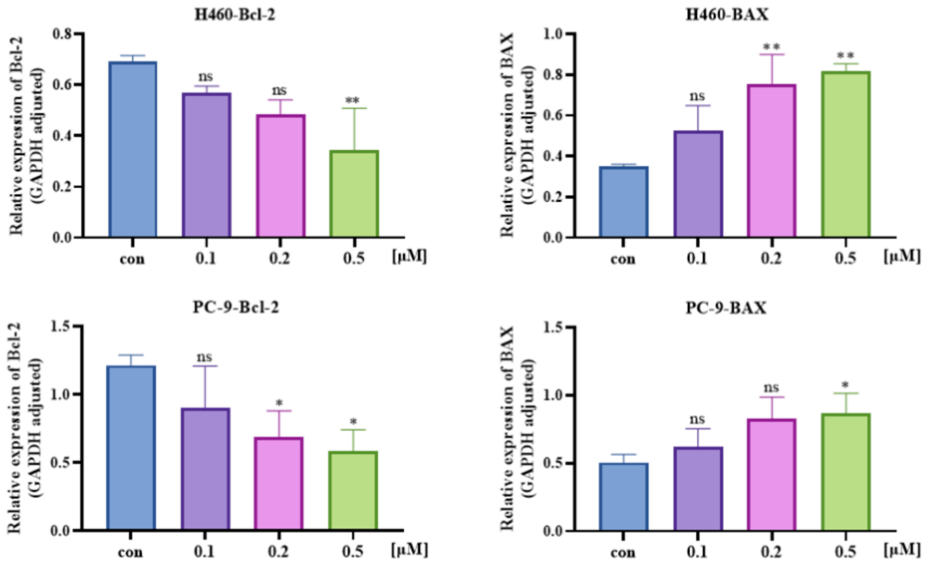

Supplementary figure 1. The relative expression of Bcl-2 and BAX

The relative expression of indicted proteins in Figure 2C with three repeats. All the data passed the Shapiro-Wilk test and Brown-Forsythe test to check the normal distribution and the homogeneity of variance. Comparisons of means among multiple groups were performed with ordinary one-way ANOVA with Tukey's multiple comparisons test. $*P < 0.05$, $**P < 0.01$.

Supplementary Figure 2

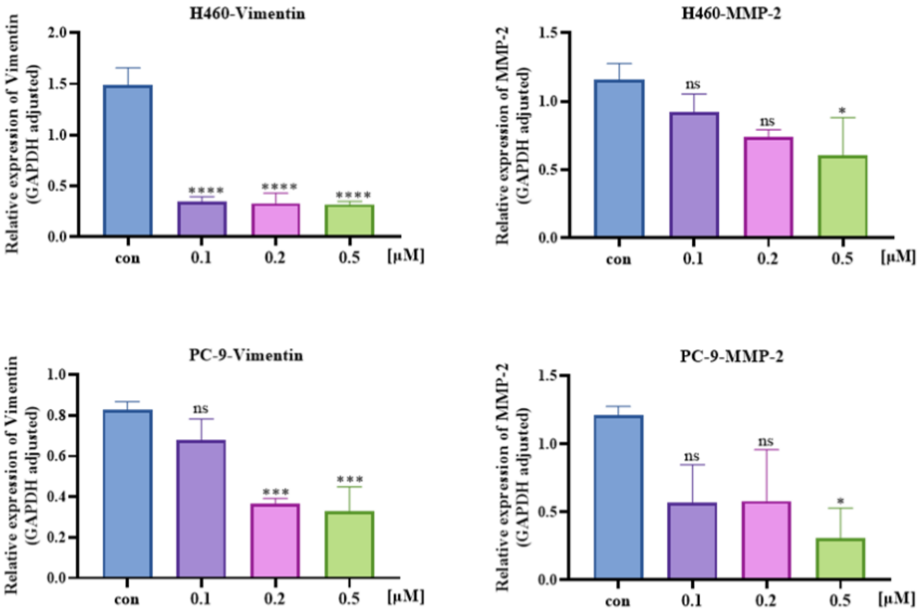

Supplementary figure 2. The relative expression of Vimentin and MMP-2

The relative expression of indicted proteins in Figure 3C with three repeats. All the data passed the Shapiro-Wilk test and Brown-Forsythe test to check the normal distribution and the homogeneity of variance. Comparisons of means among multiple groups were performed with ordinary one-way ANOVA with Tukey's multiple comparisons test. *P < 0.05, ***P < 0.001, ****P < 0.0001.

Supplementary Figure 3

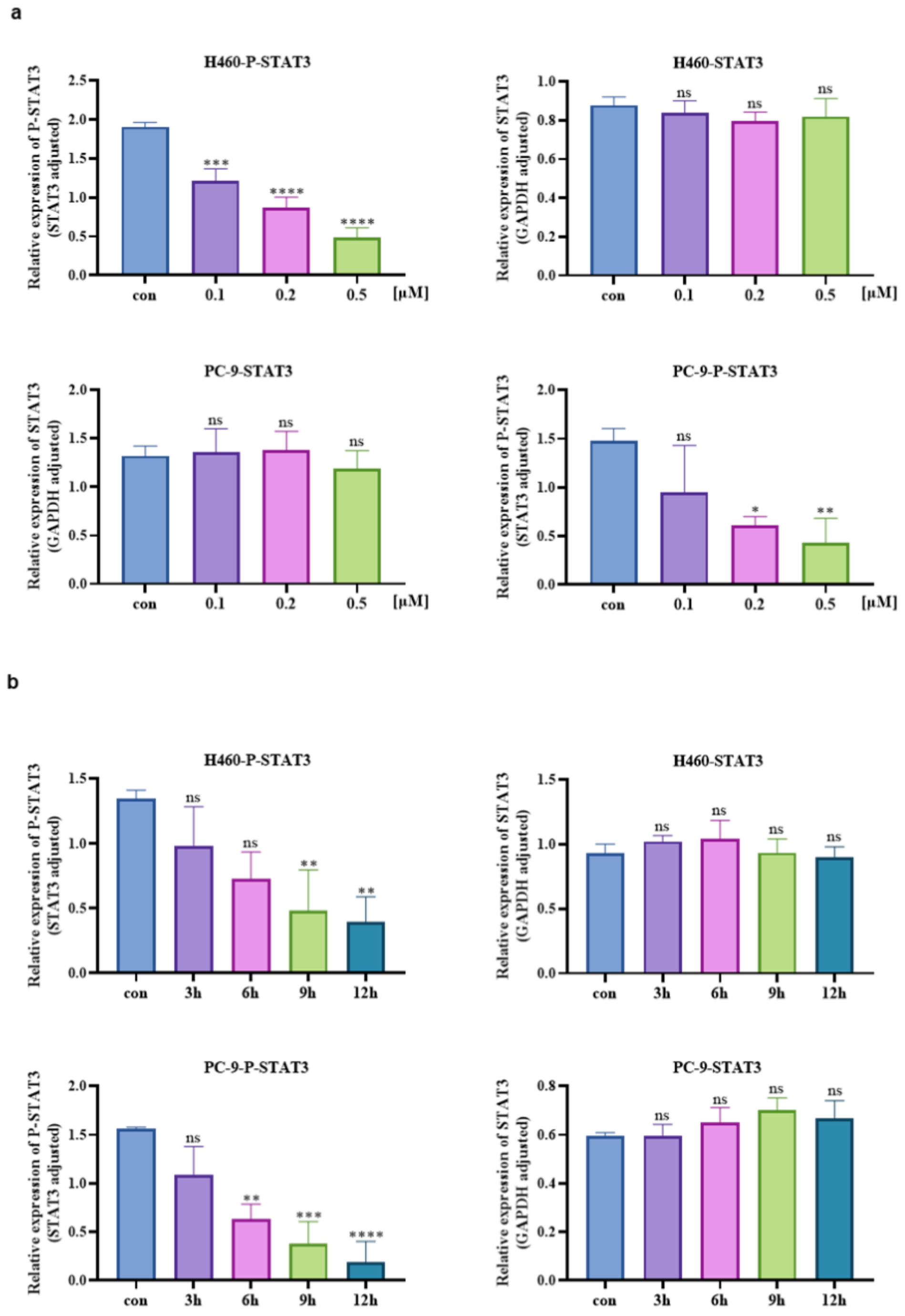

Supplementary figure 3. The relative expression of P-STAT3 and STAT3

(a) The relative expression of indicted proteins in Figure 4b with three repeats. (b) The relative expression of indicted proteins in Figure 4c with three repeats. All the data passed the Shapiro-Wilk test and Brown-Forsythe test to check the normal distribution and the homogeneity of variance. Comparisons of means among multiple groups were performed with ordinary one-way ANOVA with Tukey's multiple comparisons test. $^{**}P < 0.01$, $^{***}P < 0.001$, $^{****}P < 0.001$.
